# miR-770-5p-induced cellular switch to sensitize trastuzumab resistant breast cancer cells targeting HER2/EGFR/IGF1R bidirectional crosstalk

**DOI:** 10.55730/1300-0152.2690

**Published:** 2024-02-05

**Authors:** Senem NOYAN, Bala GÜR DEDEOĞLU

**Affiliations:** Biotechnology Institute, Ankara University, Ankara, Turkiye

**Keywords:** Bidirectional molecular crosstalk, breast cancer, miR-770-5p, tamoxifen, trastuzumab

## Abstract

**Background/aim:**

Studies highlighted the bidirectional crosstalk between the HER family members in breast cancer as resistance mechanism to anti-HER agents. Cross-signaling between HER2/EGFR and ER/IGF1R could play role in the development of resistance to therapeutics hence stimulating cell growth. To overcome this resistance, combined therapies targeting both pathways simultaneously have been proposed as an effective strategy. The involvement of miRNAs in resistance of targeted therapies like trastuzumab was demonstrated in recent studies. Hence the regulation of miRNAs in resistance state could reverse the cell behaviour to drugs. Previously we found that overexpression of miR-770-5p downregulated AKT and ERK expression through HER2 signaling and potentiated the effect of trastuzumab. In this study we examined the impact of miR-770-5p on trastuzumab resistance.

**Materials and methods:**

Cells were treated with tamoxifen or trastuzumab to examine their role in bidirectional crosstalk. The molecule mechanism of miR-770-5p on HER2/EGFR/IGF1R bidirectional crosstalk was explored by western blot. The expression of miR-770-5p in trastuzumab resistant cells was examined by q-PCR. To investigate the effect of miR-770-5p on cancer cell proliferation in trastuzumab resistance state, resistant cells were analyzed by iCELLigence real-time cell analyzer.

**Results:**

miR-770-5p expression was significantly downregulated in trastuzumab-resistant BT-474 and SK-BR-3 cells. Overexpression of miR-770-5p sensitized the resistant cells to trastuzumab, as evidenced by reduced cell proliferation and increased cell viability. Additionally, in resistant cells, increased expression and activation of EGFR and IGF1R were observed. However, miR-770-5p overexpression resulted in decreased phosphorylation of AKT and ERK, indicating its suppressive role in EGFR/HER2 signaling. Furthermore, miR-770-5p downregulated the expression of IGF1R and mTOR, suggesting its involvement in regulating the escape signaling mediated by IGF1R in resistance.

**Conclusion:**

In conclusion, our findings demonstrate the critical role of miR-770-5p in regulating bidirectional crosstalk and overcoming trastuzumab resistance in breast cancer cells. These results highlight the potential of miR-770-5p as a therapeutic target to improve the efficacy of targeted therapies and address resistance mechanisms in breast cancer.

## 1. Introduction

Breast cancer is a multifactorial and complex disease with genetic and epigenetic components, which exhibits different biological behaviors and treatment sensitivities according to molecular variabilities (Butti et al., 2018). Breast cancer is divided into different subtypes according to the presence of receptors in the clinic (Dai et al., 2015; Dong et al., 2021). Estrogen receptor (ER+) is expressed in approximately 70% of breast cancer cases (Clark et al., 1984; Chang, 2012; Tryfonidis et al., 2016). ER takes a prominent part in the development of breast cancer because it increases the expression of cyclin D1, Myc, Bcl-2, and VEGF, which play an important role in cell cycle, cell survival, and stimulation of angiogenesis (Eeckhoute et al., 2006).

For most patients with ER+ breast cancer, the primary treatment choice is endocrine therapy. Tamoxifen, a selective estrogen receptor modifier (SERM), has been the preferred option for adjuvant therapy ever since its discovery in 1970. Tamoxifen works by blocking the transcriptional activity of estrogen receptors through direct binding (Cole et al., 1971). However, despite its widespread use, patients can develop resistance to tamoxifen therapy either from the start (de novo resistance) or during the course of treatment (acquired tamoxifen resistance) ( Ring and Dowsett, 2004; Massarweh et al., 2008; Musgrove and Sutherland, 2009). Various factors are thought to contribute to tamoxifen resistance. These factors include alterations in ER signaling, interactions between ER and growth factor receptors (GFRs), decreased levels of ER expression, the regulation of specific GFRs, inactivation of PTEN within the PI3K/AKT/mTOR pathway, and the induction of NFκB signaling (Shou et al., 2004; Hosford and Miller, 2014).

Amplification of HER2 defines another subtypes of breast cancer. Overexpression of HER2 causes the activation of growth factor signaling pathways, which makes it oncogenic in breast cancer. Both genetic and pharmacological approaches have determined that HER2 is required for tumorigenesis in breast cancer models. Trastuzumab (Herceptin) is a recombinant monoclonal antibody which shows its anti tumor activity by binding to the extracellular region of HER2 in HER2-positive breast cancer (Pegram et al.; 2000; Cho et al., 2003; Hudis, 2007). While the use of trastuzumab has shown clinical benefits, the occurrence of both de novo (existing from the start) and acquired clinical resistance is on the rise (Ahmad et al., 2014; Vu et al., 2014; Luque Cabal et al., 2016). Consequently, gaining a comprehensive understanding of the molecular mechanisms behind clinical resistance to trastuzumab is essential for the development of more potent and targeted therapies (Pohlmann et al., 2009; Baselga et al., 2012; Swain et al., 2015). Investigating the mechanisms responsible for tamoxifen or trastuzumab resistance is important for developing next-generation targeted therapies against breast cancer.

The primary pathways involved in stimulating cell growth in breast cancer are the ER and HER2/EGFR signaling pathways (Krishnamurti and Silverman, 2014; Nagini, 2017; Yip and Papa, 2021). Therapeutic strategies targeting these pathways have shown success, but resistance mechanisms have emerged during both endocrine and antibody therapies, reducing their effectiveness (Ring and Dowsett, 2004; Schiff et al., 2004; Robinson et al., 2013). Research, both in preclinical and clinical settings, has focused on understanding the mechanisms behind this resistance, which is linked to a complex interplay between ER and HER2/EGFR (Arpino et al., 2008; Giuliano et al., 2013; Mazumder et al., 2021). When one pathway, such as ER, is blocked, it triggers the activation of the other pathway, like HER2/EGFR, allowing cancer cells to evade treatment-induced cell death and develop resistance (Wang et al., 2011; Giuliano et al., 2015). To combat this type of resistance, a promising approach is using combination therapies that target both pathways simultaneously. Additionally, there is another identified synergistic interaction between ER and IGF1R that promotes cell growth (Fagan and Yee, 2008).

While genetic changes resulting from drug treatment have limited evidence, several studies have demonstrated significant epigenetic modifications in drug-resistant cancer cells. In addition to extensively studied mechanisms of cancer drug resistance, recent research has associated the development of drug resistance in cancer with changes in microRNA (miRNA) expression. MiRNAs are a class of small noncoding RNA molecules, typically 17–22 nucleotides long, that regulate gene expression at the posttranscriptional level (Bartel, 2004; Cui et al., 2006; Y. Huang et al., 2011). Given the increasing identification of miRNAs in drug resistance, there is growing interest in utilizing miRNA-based approaches for breast cancer treatment (Ward et al., 2013; Ma et al., 2015; Young et al., 2017; Noyan et al., 2019).

Despite the well-defined crosstalk mechanisms mentioned earlier, there is a gap in understanding the role of miRNAs in ER-HER2 and ER-IGFR crosstalk. Therefore, further research is needed to comprehensively investigate the wide-ranging effects of individual dysregulated miRNAs to fully grasp the intricate network of feedback mechanisms and interactions between pathways. In a previous study, we examined the global expression profiles of miRNAs in breast cancer cell lines sensitive to tamoxifen and trastuzumab, identifying miR-770-5p as a common responsive miRNA after treatment with both drugs (Noyan et al., 2019). However, it remains to be determined whether miR-770-5p plays a role in the bidirectional crosstalk responsible for tamoxifen or trastuzumab responsiveness in breast cancer cells. Consequently, our current study delves into understanding the regulatory function of miR-770-5p in this bidirectional crosstalk.

## 2. Methods

### 2.1. Cell lines and reagents

Human breast carcinoma cell lines BT-474 (RRID:CVCL_0179) and SK-BR-3 (RRID:CVCL_0033) were purchased from the American Type Culture Collection. Tamoxifen was purchased from TOCRIS (Minneapolis, MN, USA). Stock solution of tamoxifen was prepared in DMSO, and stored at −20 °C. We thank Prof. Dr. Hakan Gürdal for the gift of trastuzumab (Herceptin).

Human breast cancer cell lines BT-474 parental and BT-474 resistant were cultured in RPMI 1640 with 10% fetal bovine serum (FBS); SK-BR-3 parental and SK-BR-3 resistant cells were cultured in McCoy’s medium with 10% FBS. Cells were maintained at 37 °C in a 5% CO_2_ incubator.

For 4-hydroxytamoxifen (4-OH-TAM, abbreviated as TAM) treatment, cells were cultured supplemented with 10% charcoal/dextrantreated FBS (Sigma).

### 2.2. Generation of trastuzumab-resistant clones from BT-474 and SK-BR-3 cells in vitro

Trastuzumab-resistant BT-474 (BT-474_R) and SK-BR-3 (SK-BR-3_R) cells were established in the culture media but supplemented with increasing concentrations of trastuzumab from 0.5 μg/mL, 2 μg/mL, 6 μg/mL, 10 μg/mL every six weeks. Protocol is previously described (Dolapçi et al., 2023).

### 2.3. Cell transfection

Cells were seeded into six-well plates at 4 × 10^5^ cells/well for overnight and transfected with miR-770-5p mimic (25 nM) (MSY0003948, Qiagen) or the corresponding negative control (SI03650318, Qiagen). All transfections were conducted using Hi-perfect Transfection Reagent (301705, Qiagen) following the manufacturer’s protocol and the cells were maintained 72 h after transfection.

### 2.4. Cell proliferation assay

BT-474 parental, BT-474 resistant, SK-BR-3 parental and resistant cells were seeded in 24-well plates overnight and then transfected with blank, miR-770-5p mimic or scrambled control (scr) (final concentration 25 nM). Then the cells were trypsinised and counted, seeded in 8-well plates (in triplicate) for an E-plate assay (ACEA) at a density of 8000 cells per well and incubated at 37 °C for 144 h with trastuzumab (10 μg/mL). Then, analyzed RTCA Data Analysis Software.

### 2.5. Western blotting

The protein concentration was determined using a Pierce™ Coomassie Plus (Bradford) Assay (23236, ThermoFisher Scientific) according to the manufacturer’s instructions. Equal amounts of protein (10 μg) were loaded on a 10% SDS-PAGE gel. The following antibodies were used in this study: anti-HER2 mouse antibody (1:1000, ab8054, Abcam), anti-IGF1R Rabbit antibody (1:1000, D23H3, Cell Signaling), anti-EGFR rabbit antibody (1:1000, sc373746, SantaCruz), anti-Akt mouse antibody (1:1000, sc8312, SantaCruz), antiphospho-Akt (Ser473) rabbit antibody (1:1000, sc7985, SantaCruz), anti-mTOR Rabbit antibody (1:1000, 7C10, Cell Signaling), anti-ERK2 rabbit antibody (1:1000, sc154, SantaCruz), phosho-ERK 1/2 (Thr 202/Tyr 204) antibody (1:1000, sc-81492, SantaCruz), anti-actin beta mouse monoclonal antibody (1:1000 dilution, 643802, Biolegend) and the secondary antibodies anti-rabbit immunoglobulin G (1:10000 dilution; 7074S, Cell Signaling) and anti-mouse immunoglobulin G (1:2500 dilution; 405306, Biolegend).

### 2.6. Data analysis

All experiments were done as at least two independent assays. For comparison of two groups, p-values were calculated with a student’s t-test. p < 0.05 was considered statistically significant in all cases.

## 3. Results

### 3.1. Characterization of tamoxifen or trastuzumab dependent bi-directional crosstalk in BT-474 cells

**Figure 1:**
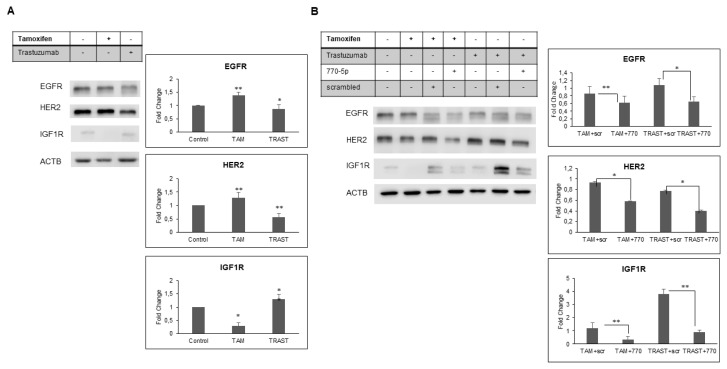
Crosstalk between EGFR-HER2-IGF1R in present tamoxifen or trastuzumab on BT-474 cells. (A) Protein levels of EGFR- HER2- IGF1R proteins in cells treated with tamoxifen (10 nM) or trastuzumab (10 mg/mL) (n = 2, *p < 0.01; **p < 0.05). (B) The effect of miR-770-5p on receptor crosstalk. miR-770-5p overexpression suppresses the crosstalk signaling between EGFR, HER2 and IGF1R. Western Blot showing the inhibitory effect of miR-770-5p on the HER2, EGFR and IGF1R expression of BT-474 cells by 10 nM tamoxifen and 10 mg/mL trastuzumab (n = 2, *p < 0.01; **p < 0.05). Western blotting was performed to detect the expression of EGFR, HER2 and IGF1R proteins using the corresponding antibodies. As a control, antibeta actin antibody was used to show that equal amounts of proteins were loaded on the gel. The error bars indicated ± standard deviation (SD). “TRAST” represents trastuzumab and “TAM” represents tamoxifen.

The ER and HER2 signaling pathways play vital roles in promoting cell proliferation in breast cancer (Cheskis et al., 2007; Seshacharyulu et al., 2012; Yarden and Pines, 2012; Saczko et al., 2017). Traditionally, therapeutic approaches have been developed to target these pathways, and they have shown success. However, the emergence of resistance mechanisms during endocrine therapy and monoclonal antibody therapy has reduced the effectiveness of these treatments. Both preclinical and clinical studies have been directed towards understanding the resistance mechanisms linked to the intricate bidirectional communication between ER and the HER family of receptors. When one pathway, like ER, is blocked, it triggers the activation of the other, such as HER2 signaling. This activation serves as a means for cancer cells to evade death and, consequently, results in resistance to therapy. A promising strategy for addressing acquired drug resistance might involve combined therapies that simultaneously target both of these pathways. Furthermore, there is another documented synergistic interaction between ER and IGFR that also promotes cell growth. This receptor crosstalk of the ER+/HER2+ BT-474 breast cancer cells treated with tamoxifen or trastuzumab was verified by western blot. Our results demonstrated that levels of EGFR and HER2 were significantly increased after treatment with 10 nM tamoxifen in BT-474 cells, whereas they were significantly reduced in these cells that were treated with 10 mg/mL trastuzumab compared with control cells (Figure 1A). Our result also showed the significant synergistic relation between the level of IGF1R and trastuzumab in BT-474 cells (Figure 1A). Taken together, these findings support that presence of bidirectional crosstalk between EGFR/HER2 and IGF1R after tamoxifen or trastuzumab treatment.

### 3.2. miR-770-5p modulates bi-directional crosstalk via targeting HER2/EGFR/IGF1R

Our previous study suggested that miR-770-5p in combination with drugs (tamoxifen or trastuzumab) could block the ER/HER2/EGFR crosstalk (Noyan et al., 2019). To investigate the role of miR-770-5p in this crosstalk we performed Western blot experiments in tamoxifen or trastuzumab treated cells. We observed that miR-770-5p overexpression inhibits EGFR, HER2 and IGF1R expression significantly compared to scrambled control (scr) in presence of tamoxifen in BT-474 cells. Moreover, the IGF1R expression was also significantly decreased upon miR-770-5p overexpression in trastuzumab treated BT-474 cells (Figure 1B). The data we gathered indicates that miR-770-5p can enhance the effectiveness of trastuzumab by regulating the elevated levels of IGF1R, which serves as an alternative mechanism for cell survival when exposed to trastuzumab. Additionally, miR-770-5p can enhance the action of tamoxifen by targeting EGFR and HER2 receptors, which are upregulated in response to tamoxifen treatment. Also, the binding sequences of miR-770-5p on the IGF1R, HER2 and EGFR were identified using the miRWalk database (Supplementary Table). These findings demonstrate the role of miR-770-5p in regulating the signaling interactions during both tamoxifen and trastuzumab treatments in BT-474 cells. These results demonstrated that miR-770-5p is critical for robust bidirectional crosstalk control during trastuzumab or tamoxifen response.

### 3.3. Restoring miR-770-5p expression increases the sensitivity to trastuzumab in resistant breast cancer cells

**Figure 2:**
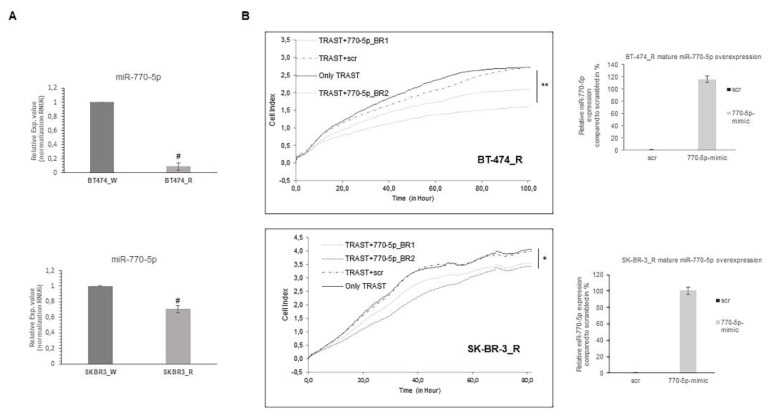
miR-770-5p was down-regulated in trastuzumab resistant cells and miR-770-5p overexpression restored trastuzumab sensitivity. (A) Trastuzumab resistance breast cancer cells show lower miR-770-5p expression. Quantitative analysis of relative expression miR-770-5p in parental and trastuzumab resistant cells (n = 2, # p < 0.01). (B) Proliferation graphs showing the effect of miR-770-5p on growth of trastuzumab resistant breast cancer cell lines. Left panel represent that miR-770-5p overexpression was consistently more effective than the only trastuzumab and trastuzumab with scrambled control (scr) group in both cells (n = 2, *p < 0.005, **p < 0.0001). Right panel shows overexpression of miR-770-5p upon transfection in breast cancer cell lines. A two-tailed t-test was used to evaluate statistical significance. The error bars indicated ± standard deviation (SD). “TRAST” represents trastuzumab and “BR” represents biological replicates.

Upon observing regulation between miR-770-5p and bidirectional crosstalk by tamoxifen and trastuzumab in drug sensitive cells, we next investigated whether miR-770-5p expression is directly involved in the regulation of trastuzumab resistant state. First, the expression level of miR-770-5p was analyzed in both BT-474 and SK-BR-3 resistant cell lines and it was found to be significantly down regulated in resistant state (Figure 2A). Cells were treated with scrambled control (scr) or miR-770-5p mimic and next we explored the effect of miR-770-5p on the growth of trastuzumab resistant cells. Proliferation analysis demonstrated that up-regulation of miR-770-5p significantly reduced cell proliferation rate compared to scrambled control (scr) in the presence of trastuzumab (Figure 2B). Resistance of the cells to trastuzumab was verified by cell viability assays. We also confirmed transfection efficiency of miR-770-5p in trastuzumab resistant cells by qRT–PCR. Accordingly, our results demonstrated that miR-770-5p-mediated regulation sensitized the resistant cells to trastuzumab.

### 3.4. Increased expression and activation of EGFR/IGF1R in the trastuzumab-resistant cells

**Figure 3:**
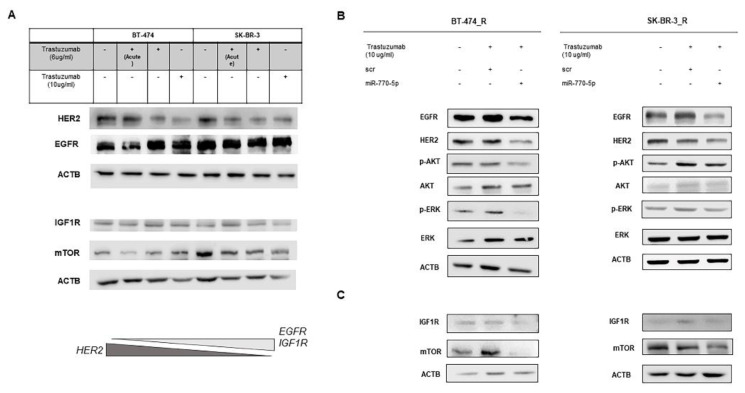
The effects of miR-770-5p on receptor crosstalk and downstream elements of p-AKT/p-ERK in trastuzumab resistant cells. (A) On acquired resistance to trastuzumab, overexpression of EGFR was occured while HER2 was decreased in both breast cancer cells. (BandC) Immunoblot analysis of protein expression levels of EGFR, HER2, IGFR and downstream element AKT, ERK, phosphorylation of AKT, ERK and mTOR in cells treated with trastuzumab, scrambled control (scr) mimic or miR-770-5p mimic. β-actin is shown as a loading control. “Acute” in the lane 2 and 6 means that BT-474 and SK-BR-3 cells were treated with 6 μg/mL of trastuzumab for 72 h.

Trastuzumab resistance is related to the enhanced signaling of the insulin-like growth factor 1 receptor (IGF1R). In BC cells, increased expression of IGF1R could enhance cell proliferation compensating HER2 down-regulation, which mediates trastuzumab resistance (Lu et al., 2001; Nahta et al., 2007). Silencing HER2 promotes cell survival via activating EGFR or IGF1R signaling (Motoyama et al., 2002; Diermeier et al., 2005; Sergina et al., 2007; Huang et al., 2010b). We hypothesized that miR-770-5p dependent regulation of EGFR/IGF1R signaling may play a role in regulating trastuzumab resistance. For this, we utilized trastuzumab resistant BT-474 and SK-BR-3 cells that were treated with increasing concentrations of trastuzumab and analyzed HER2, EGFR, IGF1R and downstream mTOR protein levels. Of importance, an increased level of EGFR was detected in trastuzumab resistant state and level of HER2 was diminished after trastuzumab treatment with gradually increased doses in acquired resistance compared with control cells (Figure 3A). Furthermore, IGF1R and mTOR expression exhibited similar results in trastuzumab resistant cells (Figure 3A). These results confirmed that up-regulation of the EGFR and IGF1R protein level could show the transactivation of bidirectional crosstalk between HER2 and EGFR/IGF1, which was shown in the literature, also in the acquired trastuzumab resistance (Balañá et al., 2001; Diermeier et al., 2005; Nahta et al., 2005; Huang et al., 2010a)

### 3.5. Effect of miR-770-5p on EGFR/HER2/IGF1R and downstream signaling in the trastuzumab-resistant cells

We next tried to gain insight into the effect of miR-770-5p overexpression on EGFR/HER2/IGF1R downstream signaling in trastuzumab resistant cells. For this, resitant cells, which were treated with trastuzumab, were transfected with scrambled control (scr) or miR-770-5p mimic. Both EGFR and HER2 were significantly reduced by the overexpression of miR-770-5p compared to srambled control. Figure 3B indicated that up-regulation of the miR-770-5p level significantly decreased the phosphorylation level of AKT and ERK in trastuzumab resistant cells, whereas it had no effect on total AKT or ERK expression levels.

Interaction of IGF1R and receptor crosstalk has been demonstrated in trastuzumab resistant breast cancer cells (Balañá et al., 2001; Nahta et al., 2005). Having demonstrated a suppressive role for miR-770-5p in EGFR/HER2 signaling, we next explored the effect of miR-770-5p on IGF1R, the one which is another escape signaling in trastuzumab resistant breast cancer cells. A similar relation was observed between the levels of IGF1R and mTOR in these cells. Upon up-regulation of miR-770-5p in resistant cells, the expression of IGF1R and mTOR was significantly reduced at both cell lines (Figure 3C). Accordingly, these results show that miR-770-5p up-regulation leads to the suppression of EGFR/IGF1R signaling in trastuzumab resistant cells.

## 4. Discussion

Breast cancer is one of the most common cancer affecting women worldwide, with a high mortality rate. Despite advances in cancer treatment, drug resistance remains a significant challenge in the management of breast cancer. Trastuzumab is a monoclonal antibody used to treat HER2-positive breast cancer. Despite its effectiveness, many patients develop resistance to trastuzumab, leading to disease progression and poorer outcomes. Understanding the mechanisms of trastuzumab resistance is therefore critical for improving patient outcomes. In recent years, microRNAs (miRNAs) have emerged as potential key regulators of trastuzumab resistance in breast cancer. miRNAs play important roles in the regulation of trastuzumab sensitivity in HER2-positive breast cancer. Dysregulated miRNAs can impact the expression and activity of key signaling pathways, leading to decreased sensitivity to trastuzumab. Targeting miRNAs that regulate trastuzumab sensitivity may provide a novel approach to overcome resistance to this important therapy in HER2-positive breast cancer. Our own and also other studies have demonstrated that miR-770-5p is downregulated in breast cancer and overexpressed miR-770-5p inhibits migration, and invasion in TNBCs where as it potentiates trastuzumab activity in HER2+ BC cells (Li et al., 2018; Noyan et al., 2019, 2021). Li et al. have indicated that overexpression of miR-770-5p is linked to the chemosensitivity of breast tumors (Li et al., 2018); however, its role in bidirectional receptor crosstalk and trastuzumab resistance have not been still determined. Here, we report a novel finding between miR-770-5p and bidirectional receptor crosstalk in breast cancer. In this study, restoring of miR-770-5p regulated EGFR/HER2/IGF1R crosstalk signaling in HER2+ breast cancer cells. Importantly, miR-770-5p re-sensitizes resistant breast cancer cells to trastuzumab.

There are several mechanisms by which breast cancer cells can develop drug resistance. One mechanism is the activation of cell signaling pathways, such as the PI3K/AKT/mTOR pathway, which promotes cancer cell survival and proliferation and reduces the effectiveness of drugs. To overcome drug resistance associated with receptor crosstalk signaling, several strategies have been proposed, including combination therapies that target multiple receptors and downstream signaling pathways. For example, dual inhibition of both the HER2 and ER receptors has been shown to be more effective than targeting either receptor alone in ER-positive, HER2-positive breast cancer. Similarly, inhibition of IGF1R signaling in combination with other targeted therapies has been proposed as a strategy to overcome resistance to ER-targeted and HER2-targeted therapies. Recent studies have also suggested that miRNAs can regulate receptor crosstalk signaling pathways, by regulating various mRNAs simultaneously, thereby impacting drug resistance in breast cancer. One example of miRNA-mediated regulation of receptor crosstalk signaling in breast cancer is the miRNA-221/222 family (Garofalo et al., 2012). These miRNAs have been shown to target the PTEN tumor suppressor gene, leading to activation of the PI3K/AKT pathway. The activation of this pathway can promote resistance to both endocrine and HER2-targeted therapies in breast cancer (Huynh et al., 2021; Di Martino et al., 2022). Additionally, miRNA-221/222 can target the ERα gene, leading to decreased sensitivity to antiestrogen therapies (Zhao et al., 2008; Di Leva et al., 2010). Another example of miRNA-mediated regulation of receptor crosstalk signaling is the miRNA-125 family (Cava et al., 2016; Peng et al., 2021). This family of miRNAs has been shown to target HER2, resulting in decreased HER2 signaling and increased sensitivity to HER2-targeted therapies. miRNAs can also regulate the crosstalk between HER2 and IGF1R signaling pathways in breast cancer. For example, miRNA-630 has been shown to target IGF1R, leading to decreased signaling through both pathways and increased sensitivity to HER2-targeted therapies (Corcoran et al., 2014).

In parallel with the aforementioned investigations, our study reveals compelling evidence that highlights the involvement of miR-770-5p in the phenomenon of trastuzumab resistance. Specifically, our findings elucidate that miR-770-5p exerts inhibitory effects on IGF1R-triggered mTOR activation, together with suppressing the activation of additional members of the HER family, including HER2 and EGFR. Based on what we found, we could suggest that miR-770-5p sensitize the resistant cells to trastuzumab via the EGFR/HER2 and IGFR also with downstream signaling. Moreover, the decreased p-AKT and p-ERK signaling supported the sensitizing effect of miR-770-5p in resistant cells. The intricate interplay mediated by miR-770-5p in this context renders it a promising and noteworthy candidate for the development of combined therapeutic interventions aimed at circumventing trastuzumab resistance.

In summary, miRNAs play important roles in the regulation of receptor crosstalk signaling and drug resistance in breast cancer. Dysregulated miRNAs can impact the sensitivity of breast cancer cells to targeted therapies by altering the expression and activity of key signaling pathways. Targeting miRNAs that regulate receptor crosstalk signaling may provide a novel approach to overcome drug resistance in breast cancer. This study sheds light on the impact of miR-770-5p-mediated bidirectional crosstalk in HER2-positive breast cancer. Our findings identify miR-770-5p as a tumor suppressor in breast tumors, acting as a regulatory switch that targets key players such as EGFR, HER2, IGF1R, as well as downstream effectors including AKT, ERK, and mTOR. Future investigations involving animal models and functional characterization are needed to further elucidate the precise role of miR-770-5p, which could potentially facilitate the restoration of its expression as a pivotal discovery in enhancing trastuzumab resensitivity in resistant breast cancer cases.

## 5. Conclusion

**Figure 4:**
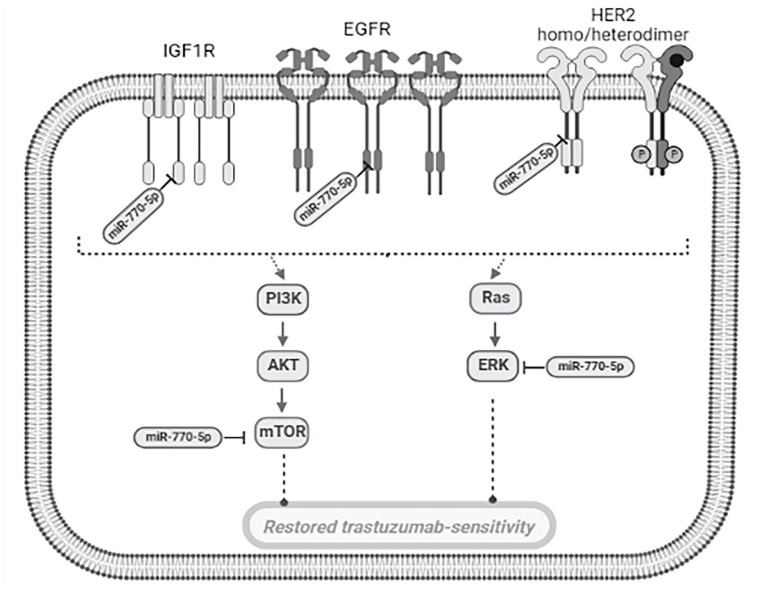
Proposed model of miR-770-5p’s role in crosstalk between EGFR, HER2, and IGF1R signaling pathways. The effect of miR-770-5p on the EGFR/HER2/IGF1R crosstalk signaling. In general, tumorigenesis of breast cancer is assumed to be associated with the PI3K and MAPK pathways and signaling these pathway complex has critical roles in cell proliferation in HER2 amplified cells. Trastuzumab can block this signaling, either by inhibiting the activity of EGFR and HER2 kinases directly or through HER2 binding at the cell surface. miR-770-5p can reduce dissociation of receptor crosstalk signaling, which increases its activity.

The findings of this study led us to propose that overexpression of miR-770-5p could restore trastuzumab sensitivity in resistant cells via regulating EGFR/HER2/IGF1R bidirectional crosstalk (Figure 4). Future in vivo studies will be valuable to address the clinical significance of miR-770-5p-based approaches to regulate the sensitivity to hormonal and targeted therapies.

## Supplementary Materials

**Supplementary Table:** In silico analysis of miR-770-5p targets.

mirnaid	refseqid	gene	symbol duplex	start	end	bindingp	energy	seed	number_of_pairings	binding_region_length	longest_consecutive_pair	position
hsa-miR-770-5p	NM_001382804	ERBB2	TCCAGTACCACGTGTCAGGGCCA#TGGCGGTGGGGACCTGACACTAGGGCTGGAG#	2508	2539	0,9230769	−28,3	0	20	31	8	CDS
hsa-miR-770-5p	NM_001382804	ERBB2	TCCAGTACCACGTGTCAGGGCCA#TGGTCTTGGGGGTGGTCTTTGGGA#	1340	1364	0,8461538	−24,7	0	20	24	9	CDS
hsa-miR-770-5p	NM_001289936	ERBB2	TCCAGTACCACGTGTCAGGGCCA#TGGCGGTGGGGACCTGACACTAGGGCTGGAG#	3688	3719	0,9230769	−28,3	0	20	31	8	CDS
hsa-miR-770-5p	NM_001289936	ERBB2	TCCAGTACCACGTGTCAGGGCCA#GCCCTGGCCGTGCTAGACAATGGAG#	863	888	0,9230769	−25	0	18	25	12	CDS
hsa-miR-770-5p	NM_001289937	ERBB2	TCCAGTACCACGTGTCAGGGCCA#GCCCTGGCCGTGCTAGACAATGGAG#	511	536	0,9230769	−25	0	18	25	12	CDS
hsa-miR-770-5p	NM_001289937	ERBB2	TCCAGTACCACGTGTCAGGGCCA#TGGTCTTGGGGGTGGTCTTTGGGA#	2168	2192	0,8461538	−24,7	0	20	24	9	CDS
hsa-miR-770-5p	NM_001289938	ERBB2	TCCAGTACCACGTGTCAGGGCCA#GCCCTGGCCGTGCTAGACAATGGAG#	812	837	0,9230769	−25	0	18	25	12	CDS
hsa-miR-770-5p	NM_004448	ERBB2	TCCAGTACCACGTGTCAGGGCCA#TGGCGGTGGGGACCTGACACTAGGGCTGGAG#	3336	3367	0,9230769	−28,3	0	20	31	8	CDS
hsa-miR-770-5p	NM_004448	ERBB2	TCCAGTACCACGTGTCAGGGCCA#GCCCTGGCCGTGCTAGACAATGGAG#	511	536	0,9230769	−25	0	18	25	12	CDS
hsa-miR-770-5p	NM_004448	ERBB2	TCCAGTACCACGTGTCAGGGCCA#GGGCTGCCAGGTGGTGCAGGGAA#	327	350	0,8461538	−25,2	0	17	23	7	CDS
hsa-miR-770-5p	NM_001005862	ERBB2	TCCAGTACCACGTGTCAGGGCCA#TGGCGGTGGGGACCTGACACTAGGGCTGGAG#	3637	3668	0,9230769	−28,3	0	20	31	8	CDS
hsa-miR-770-5p	NM_001005862	ERBB2	TCCAGTACCACGTGTCAGGGCCA#GCCCTGGCCGTGCTAGACAATGGAG#	812	837	0,9230769	−25	0	18	25	12	CDS
hsa-miR-770-5p	NM_001005862	ERBB2	TCCAGTACCACGTGTCAGGGCCA#GGGCTGCCAGGTGGTGCAGGGAA#	628	651	0,8461538	−25,2	0	17	23	7	CDS
hsa-miR-770-5p	NM_001291858	IGF1R	TCCAGTACCACGTGTCAGGGCCA#GGCCCCGGCCCTGGGGTGCTGGT#	5021	5044	0,8461538	−29,5	1	18	23	8	CDS
hsa-miR-770-5p	NM_001291858	IGF1R	TCCAGTACCACGTGTCAGGGCCA#GGACCAGGGCTGTGGTGCTGGC#	7268	7290	0,8461538	−29,5	1	18	22	14	3UTR
hsa-miR-770-5p	NM_001291858	IGF1R	TCCAGTACCACGTGTCAGGGCCA#CCCTGAGGCAGCAGGAGCTGGTGTGTACTGGAG#	9393	9426	1	−27,4	1	19	33	8	3UTR
hsa-miR-770-5p	NM_000875	IGF1R	TCCAGTACCACGTGTCAGGGCCA#GGCCCCGGCCCTGGGGTGCTGGT#	5024	5047	1	−29,5	1	18	23	8	CDS
hsa-miR-770-5p	NM_000875	IGF1R	TCCAGTACCACGTGTCAGGGCCA#GGACCAGGGCTGTGGTGCTGGC#	7271	7293	1	−29,5	1	18	22	14	3UTR
hsa-miR-770-5p	NM_000875	IGF1R	TCCAGTACCACGTGTCAGGGCCA#CCCTGAGGCAGCAGGAGCTGGTGTGTACTGGAG#	9396	9429	1	−27,4	1	19	33	8	3UTR
hsa-miR-770-5p	NM_001346897	EGFR	TCCAGTACCACGTGTCAGGGCCA#GGCCTTGCCGCAAAGTGTGTAACGGAATAGGTATTGGT#	1117	1155	1	−24,4	1	18	38	8	CDS
hsa-miR-770-5p	NM_001346900	EGFR	TCCAGTACCACGTGTCAGGGCCA#GGCCTTGCCGCAAAGTGTGTAACGGAATAGGTATTGGT#	1023	1061	1	−24,4	1	18	38	8	CDS
hsa-miR-770-5p	NM_201282	EGFR	TCCAGTACCACGTGTCAGGGCCA#GGCCTTGCCGCAAAGTGTGTAACGGAATAGGTATTGGT#	1252	1290	1	−24,4	1	18	38	8	CDS
hsa-miR-770-5p	NM_201283	EGFR	TCCAGTACCACGTGTCAGGGCCA#GGCCTTGCCGCAAAGTGTGTAACGGAATAGGTATTGGT#	1252	1290	1	−24,4	1	18	38	8	CDS
hsa-miR-770-5p	NM_201284	EGFR	TCCAGTACCACGTGTCAGGGCCA#GGCCTTGCCGCAAAGTGTGTAACGGAATAGGTATTGGT#	1252	1290	0,9230769	−24,4	1	18	38	8	CDS
hsa-miR-770-5p	NM_201284	EGFR	TCCAGTACCACGTGTCAGGGCCA#GGCCCCCTGACTCCGTCCAGTATTGA#	199	225	0,8461538	−23,5	1	18	26	6	5UTR
hsa-miR-770-5p	NM_005228	EGFR	TCCAGTACCACGTGTCAGGGCCA#GGCCTTGCCGCAAAGTGTGTAACGGAATAGGTATTGGT#	1252	1290	1	−24,4	1	18	38	8	CDS
